# Ketamine Use for Cancer and Chronic Pain Management

**DOI:** 10.3389/fphar.2020.599721

**Published:** 2021-02-02

**Authors:** Clayton Culp, Hee Kee Kim, Salahadin Abdi

**Affiliations:** ^1^ McGovern Medical School, University of Texas Health Science Center Houston (UTHealth), Houston, TX, United States; ^2^ Division of Anesthesiology, Department of Pain Medicine, Critical Care and Pain Medicine, The University of Texas MD Anderson Cancer Center, Houston, TX, United States

**Keywords:** analgesia, chronic pain, cancer pain, ketamine, ketamine infusion, mechanism of action, neuropathic pain

## Abstract

Ketamine, an N-methyl-D-aspartate receptor antagonist, is widely known as a dissociative anesthetic and phencyclidine derivative. Due to an undesirable adverse event profile when used as an anesthetic it had widely fallen out of human use in favor of more modern agents. However, it has recently been explored for several other indications such as treatment resistant depression and chronic pain. Several recent studies and case reports compiled here show that ketamine is an effective analgesic in chronic pain conditions including cancer-related neuropathic pain. Of special interest is ketamine’s opioid sparing ability by counteracting the central nervous system sensitization seen in opioid induced hyperalgesia. Furthermore, at the sub-anesthetic concentrations used for analgesia ketamine’s safety and adverse event profiles are much improved. In this article, we review both the basic science and clinical evidence regarding ketamine’s utility in chronic pain conditions as well as potential adverse events.

## Introduction

Ketamine, a phencyclidine derivative, was first synthesized in the 1960s as a short-acting alternative to phencyclidine which also demonstrated lower occurrence rates of emergence delirium (Domino, 2010). The drug gained FDA approval for use as a sole or combine general anesthetic agent in 1970 with administration intravenously or intramuscular (Rosenbaum et al., 2020). While not currently FDA approved, there has been significant clinical evidence that suggests administration of ketamine at sub-anesthetic doses provides significant analgesic effects with limited side effects (Kronenberg, 2002). Indeed, intravenous ketamine infusions for chronic refractory pain are within the guidelines of the American Society of Anesthesiology (Cohen et al., 2018). Likewise, growing evidence supports alternative modes of delivery of ketamine in analgesia (Kronenberg, 2002). The purpose of this review is to highlight the current understanding of ketamine use in chronic pain management especially as it relates to cancer pain.

## Chemistry, Pharmacology, and Pharmacogenomics

### Ketamine Pharmacokinetics

**FIGURE 1:**
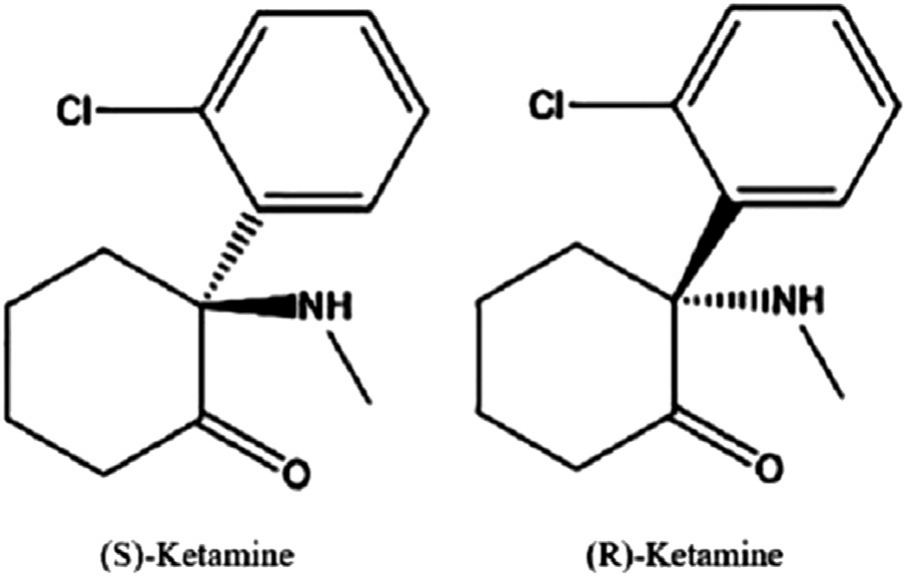
Chemical structural of the two ketamine enanitomers.

The proper IUPAC chemical name for ketamine is 2-(2-chlorophenyl)-2-(methylamino)-cyclohexanone ketamine. It consists of two optical enantiomers, both of which can be seen in Figure 1 **,** due to an asymmetric C2 carbon atom. It exhibits non-polarity and is therefore highly lipid soluble. While both the racemate mixture and (S)-Ketamine are clinically available as water soluble powders, there are currently no commercial (R)-Ketamine solutions. This is likely due to the observation that (S)-Ketamine is several times more potent at producing anesthetic and analgesic effects (Sinner and Graf, 2008).

In a study of healthy patients, the volume of distribution is estimated as 380–650 L/70 kg while the oral bioavailability was found to be in the range of 7–20%. The low bioavailability is due to a high first pass metabolism evidenced by the fact that clearance of ketamine was 83–121 L/h/70 kg which is in the range of liver blood flow. It can therefore be deduced that conditions such as cirrhosis which reduce hepatic blood flow will lower ketamine clearance (Dahan et al., 2011).

Initial metabolism of ketamine occurs via oxidation by the cytochrome P450 family of enzymes, most notably CYP3A4 and CYP2B6, which produces norketamine, 6-hydroxyketamine, and 4-hydroxyketamine. Norketamine retains pharmacologic activity and is considered the major metabolite as approximately 80% of ketamine is metabolized to this form. The remaining 20% is metabolized by one of two pathways to 4- or 6-hydroxyketamine before subsequently metabolized to hydroxy-norketamine (Kharasch and Labroo, 1992; Hijazi and Boulieu, 2002).

### Ketamine Drug Interactions

Ketamine has been shown to have interactions on the metabolism and activity of many other drugs that must be considered when used clinically. Of special note is ketamine’s reliance on the cytochrome P450 family of enzymes for its metabolism and degradation.

Rifampin, a well-known inducer of the CYP3A subfamily of enzymes, greatly reduces the area under the curve (AUC) for ketamine plasma concentration (14% IV, 86% Oral) and reduced the maximum plasma concentration after oral ketamine by 81% while not significantly shortening half-life of the drug (Peltoniemi, et al., 2012a).

In contrast, patients given Clarithromycin demonstrated a 2.6-fold increase in maximum Ketamine plasma concentration due to inhibition of the enzyme CYP3A resulting in a self-reported increase in drug efficacy (Hagelberg et al., 2010). Grapefruit juice, another Cytochrome P450 inhibitor, increased the mean AUC for ketamine plasma concentration by 3-fold and increased maximum plasma concentration of ketamine 2.1-fold while increasing half-life 21%. The half-life and maximum concentration of ketamine’s major metabolite norketamine were not significantly changed (Peltoniemi, et al., 2012b). In both studies investigating inhibition of ketamine metabolism no adverse events were noted, likely due to the use of a low dosage, however based on clinical experience it is presumed that adverse events would be more prevalent in patients taking ketamine and exposed to clarithromycin or grapefruit juice.

In addition to effects on the P450 family of enzymes, ketamine also inhibits many human UDP-glucuronosyltransferase (UGT) enzymes with the exception of UGT1A4. UGT2B4, UGT2B7, and UGT2B15 were inhibited with IC50’s less than <100 μM ketamine. Of specific interests, inhibition of UGT2B7 is responsible for metabolism of Morphine and Codeine. At anesthetic doses, inhibition of this enzyme was clinically significant for metabolism for Morphine and Codeine while at analgesic doses the inhibition was only clinically significant for Codeine metabolism (Uchaipichat et al., 2011).

Another important drug interaction occurs with benzodiazepines which are frequently used to reduce dissociative symptoms of ketamine. Pre-treatment of surgical patients with diazepam results in higher plasma concentrations of ketamine and decreased ketamine clearance (Domino et al., 1984). Pre-treatment with diazepam also reduced the cardiac stimulation of ketamine and reduced initial infusion dose required compared to no premedication (Idvall et al., 1983). Haloperidol, an antipsychotic, has also been shown to modulate the CNS effects of sub-anesthetic ketamine and reduce ketamine-induced cognitive impairment (Krystal et al., 1999).

### Ketamine Pharmacogenomics

With the increasing field-wide focus on personalized medicine, much work has also been done to understand how genetics can impact the metabolism and clearance of ketamine. Due to the wealth of information and availability of genetic information the cytochrome P450 family, especially CYP3A4 and CYP2B6 enzymes, have been the focus of much research.

One study surveyed the catalytic activity of thirty-eight CYP2C9 alleles on ketamine metabolism to norketamine. Compared with 2C9*1, three alleles (2C9*40, *49 and *51) demonstrated dramatically increased intrinsic clearance (1.2-fold–3.75-fold); four subtypes (2C9*27, *31, *41, and *56) exhibited no significant change on enzyme activity. The remaining 31 alleles expressed different degrees of reduction compared with wild type (Zheng et al., 2017).

Another *in vitro* study demonstrated that genetic variations in the CYP2B6 gene caused significant differences in plasma clearance of ketamine as well as in the ratio of norketamine: ketamine metabolic ratios. Specifically, those with the CYP2B6*6/*6 genotype exhibited a significantly lower plasma clearance of ketamine and norketamine: ketamine ratio when compared with both the CYP2B6*6/*1 and CYP2B*1/*1 genotypes (*p* < 0.05) (Li et al., 2013).

With this information, CYP2B6 genotypes were identified in 49 chronic pain patients who received 24 h continuous subcutaneous infusions of ketamine. Steady-state plasma concentrations of ketamine and norketamine were determined using High Performance Liquid Chromatography (HPLC). Patients with the CYP2B6*6 allele demonstrated a substantial decrease in steady-state ketamine plasma clearance (Li et al., 2015). This decreased ketamine clearance is clinically significant as it could potentially be associated with a higher incidence of ketamine-induced adverse events.

The importance of the CYP2B6 gene was confirmed by another study which demonstrated that per molecule the enzymatic activity of the CYP2B6 gene product was higher than that of CYP3A4 or CYP2C9 when comparing N-demethylation of ketamine to norketamine. However, the same study demonstrated that when adjusted for the average relative content of these three enzymes in human hepatocytes CYP3A4 is the principle enzyme of ketamine metabolism at therapeutic concentrations due to is ∼30-fold higher abundance (Hijazi & Boulieu, 2002). Unfortunately, no research has yet been published on the effect of different CYP3A4 alleles on ketamine metabolism and clearance. This could be an area for ketamine pharmacogenomic research moving forward.

## Mechanism of Action

### General Mechanism of Action

Ketamine’s mechanism of action is widely regarded as one of pharmacology’s most complex due to its interaction with multiple binding sites including NMDA receptors, opioid receptors, Na channels, and L-type calcium channels (Kohrs and Durieux, 1998).

**FIGURE 2:**
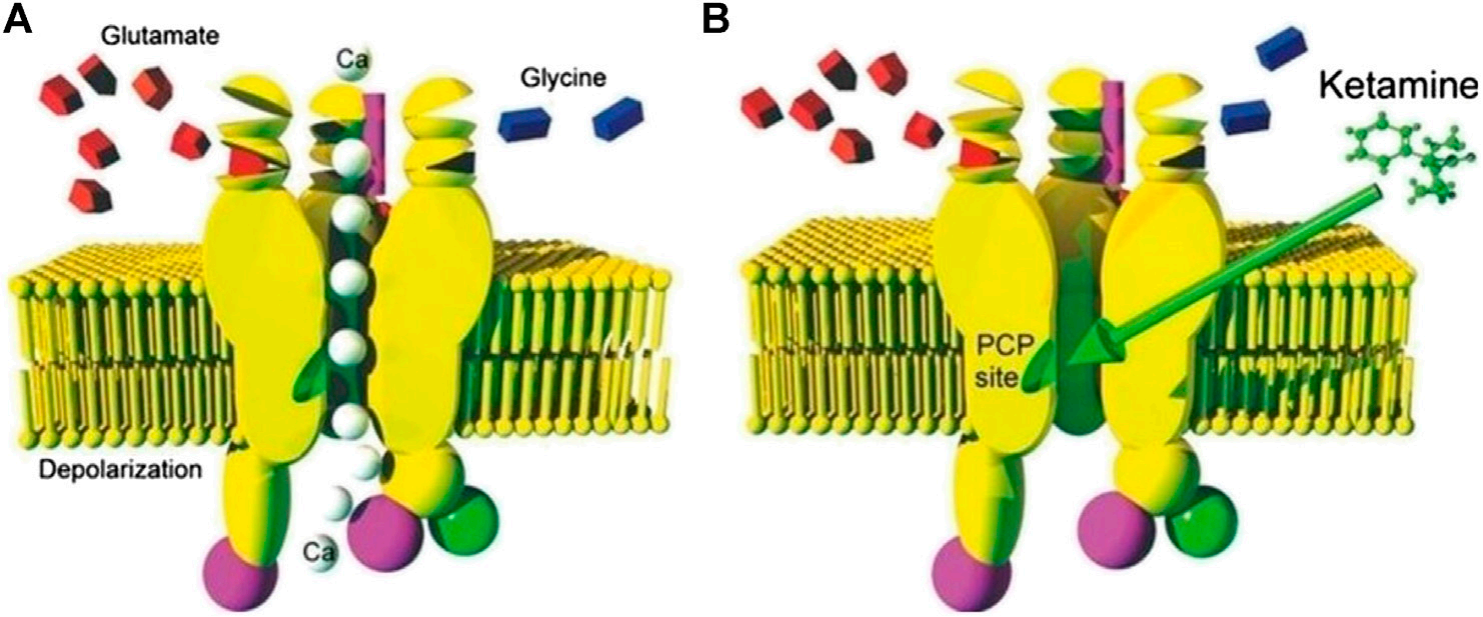
**(A)** Depiction of an NMDA Receptor in the open state and allowing passage of calcium ions due to binding of glutamate and or/glycin **(B)** An image of an NMDA receptor blockaded by binding of ketamine due to binding of the drug at the PCP binding site Mion and Villevieille (2013).

The primary anesthetic effect of ketamine is associated with NMDA receptor blockade which utilizes an uncompetitive mechanism with a relatively long knock-off time. Blockade of NMDA receptors disrupts normal excitatory glutamate signaling in the CNS and is associated with many anesthetic agents. A depiction of ketamine’s action on the NMDA receptor can be seen in Figure 2 (Mion and Villevieille, 2013)**.**

Besides NMDA receptors, ketamine also exhibits high affinity for the dopamine D2 and serotonin 5-HT_2_ binding sites and significantly inhibits their uptake. Affinity of ketamine for the 5-HT site is equal to that of ketamine for NMDA receptors. Affinity for the D2 site is 1/3rd of that (Kapur and Seeman, 2002). The fact that the binding affinities are similar to that of the NMDA receptor means significant binding of ketamine to D2 and 5-HT_2_ receptors will occur at clinically relevant drug concentrations and may be responsible for some of ketamine’s clinical effects. Indeed, 5-HT antagonists such as methysergide have been shown to antagonize ketamine’s analgesic effect; indicating that serotonergic pathways are likely involved in ketamine analgesia (Crisp et al., 1991).

Ketamine has also been shown to bind to all types of opioid receptors with the highest affinity for mu receptors, followed by kappa, and finally delta receptors (Smith et al., 1998; Sleigh et al., 2014). While opioid receptors are often associated with analgesia, ketamine’s interactions with these receptors are not believed to produce a significant analgesic effect. This is due to ketamine’s affinity for the opioid receptors being at least ten-fold less than for that of NMDA receptors (Smith et al., 1998). Additionally, ketamine’s analgesia is not reversed by administration of mu opioid receptor agonists such as naloxone which suggests the mu receptor binding plays a minimal (if any) roll in ketamine analgesia (Bansinath et al., 1992).

Muscarinic receptors are antagonized by ketamine although with 10 to 20 fold less affinity than NMDA receptors. The inhibitory effect of ketamine on the muscarinic system is believed to play a role in some peripheral side effects of the drug such as increased sympathetic tone, pupillary dilation, and bronchodilation. It is also hypothesized that the anti-muscarinic effect of ketamine may be partly responsible for ketamine-induced amnesia as the muscarinic pathways have been demonstrated to play important roles in memory, consciousness, and learning (Durieux, 1995).

### Inhibition of Astrocyte Activation

One of the more recent uses of ketamine treatment has been in the targeted treatment of neuropathic pain. Ketamine is believed to inhibit the development and lessen the severity of neuropathic pain conditions by inhibiting the inflammatory signaling pathway of some glial cell types which is associated with development of pain states.

In mouse models astrocyte activation (as measured by increased GFAP) corresponded to development of neuropathic pain behavior. Meanwhile, administration of minocycline (microglial activation inhibitor) and pentoxifylline (cytokine release inhibitor) reduced CCI-related hypersensitivity (Mika et al., 2009). Likewise, activation of glial cells results in release of pro-inflammatory cytokines, similar to those seen with a spinal infection, that can be correlated with pathological pain (Watkins et al., 2001).

The increased use ketamine in neuropathic has been fueled by research indicating that ketamine may not only provide analgesia for these conditions but also stop the development and advancement of neuropathic pain at its source. For example, a mouse spinal nerve ligation (SNL) model demonstrated that ketamine administration inhibits astrocyte activation and down-regulates expression of GFAP protein (X. Mei et al., 2009).

Ketamine may also play a role in decreasing neuronal inflammation by inhibiting cytokine release as ketamine reduces LPS-induced TNF-a production in mixed glial cell cultures (Shibakawa et al., 2005).

Other models have shown that administration of S-ketamine entirely inhibited pharmacologically induced tactile allodynia via inhibition of Voltage-Gated Calcium Dependent (BK) Potassium Channels leading to decreased mIL-1B and BDNF secretion and reduced glial activation. Another known inhibitor of this channel, charybdotoxin, was shown to significantly reduce nerve injury induced tactile allodynia in mice (Hayashi et al., 2011).

### Ketamine and Opioid-Induced Hyperalgesia (OIH)

While the exact mechanism of Opioid Induced Hyperalgesia (OIH) is unknown, one of the leading theories is overactivation and stimulation of the NMDA-receptor. OIH is more commonly observed following high-dose opioid administration such as that used for refractory cancer pain. This proposed mechanism would explain why NMDA Receptor modulators such as ketamine are effective in treating the condition (Lee et al., 2011).

One study proving this efficacy demonstrated that compared to those who underwent anesthesia with high-dose remifentanil only, patients receiving high-dose remifentanil and intra-operative ketamine infusion experienced less post-operative hyperalgesia as measured by pain rating at 6 and 24 h and cumulative fentanyl dose before release (Choi et al., 2015). In another study, patients on a ketamine infusion of 2 mg/kg/min following lumbar spinal fusion had significantly lower total fentanyl consumption than a 1 mg/kg/min dose and control patients. The incidence of adverse events was not significantly different between groups (Kim et al., 2013).

Together, these studies indicate that ketamine may counteract OIH and thereby decreased total daily oral morphine equivalent required for pain management. Clinically, this information has been applied by using ketamine as an agent to reduce total opioid usage perioperatively, treat opioid induce neurotoxicity, and to control pain that has stopped responding to opioid escalation (Gharaei et al., 2013; Morley, 2003; Winegarden et al., 2016). Given the current opioid addiction epidemic and adverse event profile of opioids the potential for ketamine to reduce the magnitude of their use should be researched further.

## Basic Science Evidence of Ketamine Efficacy

### Chemotherapy-Induced Neuropathic Pain

Due to the frequency of chemotherapy-induced neuropathic pain in cancer patients, an established animal model for this pain condition (paclitaxel-induced neuropathy) was used to determine the potential efficacy of ketamine. In two separate studies systemic ketamine injection ameliorated pain behaviors including mechanical allodynia and thermal hyperalgesia rats (Pascual et al., 2010; Hwang et al., 2012). Interestingly, one study found that a higher dose was required to significantly reduce pain thresholds in male rats when compared to their female counterparts (Hwang et al., 2012).

### Nerve-Injury-Induced Neuropathic Pain

Nerve injury-induced neuropathic pain animal models include spinal nerve ligation (SNL), chronic constriction injury (CCI), spared nerve injury (SNI), constriction of the infraorbital nerve, and complete spinal cord transection in animals including rat and mouse.

The single systemic injection of ketamine reduced several nociceptive pain behaviors including mechanical allodynia and hyperalgesia, cold allodynia, spontaneous pain in SNL model in rat at dose of 0.01, 1, 5, 10 mg/kg. However, high doses (25, 50 mg/kg) produced adverse effects including motor impairment (Qian et al., 1996; Suzuki et al., 2001). In addition, repeated systemic injection or systemic infusion of ketamine also reduced mechanical allodynia in SNL in rats at dose of 3, 10 mg/kg daily or 40 mg/kg/day (Kwon et al., 2014; Schwenk et al., 2016). In the SNL model in rat, intrathecal injection of ketamine reduced mechanical allodynia at a dose of 10, 30, 100, 300, 1,000 μg/kg, which means that the spinal cord was major site of action of ketamine (Burton et al., 1999; Cohen et al., 2009; Mei et al., 2010; Mei, et al., 2011a; Mei, et al., 2011b).

In the SNI model, repeated injection or systemic infusion of ketamine reduced mechanical and cold allodynia for several weeks at dose of 9 mg/kg/day for 5 days or 3 mg/kg/h for 3 h daily, total for 5 days (Cohen et al., 2004; Swartjes et al., 2011). However, the single systemic injection did not produce analgesic effect at a dose of 10 mg/kg (Cohen et al., 2006).

In the CCI model, systemic injection of ketamine reduced heat hyperalgesia and mechanical allodynia for several hours at a dose of 30, 40, and 50 mg/kg (Vissers et al., 2006; Nina Dimitrova et al., 2019). Its ED25 was 31.8 mg/kg by single subcutaneous injection (Pelissier et al., 2003).

### Diabetic Neuropathic Pain

Another condition which frequently manifests with neuropathic pain is chronic diabetes. In mice, this can be modeled with high-dose injection of streptozotocin which selectively kills pancreatic beta cells through DNA alkalization.

In two studies of streptozotocin-induced diabetic neuropathy, systemic infusion of ketamine at 20 mg/kg/day for 5 days reduced heat and mechanical hyperalgesia for several weeks following treatment (Mak et al., 2015; Claudino et al., 2018). In one of the studies, ketamine-treated mice also demonstrated some opioid-sparing effects for up to 6 weeks with an increased sensitivity to acute nociceptive effects of morphine (Mak et al., 2015).

### Other Neuropathic Pain

While three of the most common neuropathic pain conditions have been discussed above, there are a multitude more neuropathic pain models available in animals to test the efficacy of ketamine. In various models including arthritic pain, distal tibia fracture and casting, ischemia and reperfusion, and herpetic pain, the systemic injection of ketamine reduced pain behaviors (Ward and Standage, 2003; Sasaki et al., 2008; Tajerian et al., 2015; Zhou et al., 2018).

In the distal tibia fracture and casting in mouse, subcutaneous injections of ketamine (2 mg/kg/day for 7 days starting week 7 after fracture) reduced allodynia for 4 weeks after its termination (Tajerian et al., 2015).

In the ischemia and reperfusion models in rat, intraperitoneal injection of ketamine (100 mg/kg) reduced mechanical and cold allodynia (Liman et al., 2015).

In herpetic pain model using inoculation of Herpes simplex virus type 1 on skin of the femur in female C57BL/6 J mice, intraperitoneal injection of ketamine (50 mg/kg) reduced allodynia on day 7 after inoculation (Sasaki et al., 2008).

The success of ketamine in treating such a wide array of neuropathic pain models is promising as it suggests that ketamine is acting to reverse/inhibit a process involved in the general development of neuropathic pain rather than a process which may be specific to a single animal model. A summary of all the discussed basic science and animal model evidence of ketamine's efficacy as an analgesic can be found in Table 1.

**TABLE 1:** Animal studies using ketamine for chronic pain.

Authors	Animal model	Ketamine treatment	Results
Chemotherapy-induced neuropathic pain			
Hwang et al. (2012)	Paclitaxel-induced neuropathic pain in rats	Intraperitoneal injection of 2 or 5 mg/kg ketamine	The lower dose of 2 mg/kg only reduced mechanical allodynia in male rats. The 5 mg/kg dose reduced mechanical allodynia in both male and female rats
Pascual et al. (2010)	Paclitaxel-induced neuropathic pain in rat	Intraperitoneal injection of ketamine (maximum 50 mg/kg)	Only the highest dose of ketamine (50 mg/kg) reduced the mechanical allodynia and thermal hyperalgesia induce by paclitaxel
Nerve injury-induced neuropathic pain			
Schwenk et al. (2016)	Spinal nerve ligation in rats	Intraperitoneal injection of 3 or 10 mg/kg ketamine daily for 4 weeks	Ketamine increased mechanical pain thresholds during weeks 2–4
Qian et al. (1996)	Spinal nerve ligation in rats	Intraperitoneal injection of 0.01.1, 25, or 50 mg/kg ketamine	Ketamine injection up to 1 mg/kg reduced nociceptive behaviors for a minimum of 15 min with the higher doses providing longer relief up to 75 min without noticeable side effects. The two highest doses (25 and 50 mg/kg) provided even longer relief but induced transient motor impairment
Suzuki et al. (2001)	Spinal nerve ligation in rats	Intravenous injection of 1, 5, 10 mg/kg ketamine	Ketamine produced a greater decrease in the noxious and innocuous mechanical evoked responses when compared to MK-801 and memantine
Kwon et al. (2014)	Spinal nerve ligation in rats	Subcutaneous infusion of 40 mg/kg/day ketamine for 7 days	Ketamine reduced mechanical allodynia after 3 days of infusions
Mei, et al. (2011a)	Spinal nerve ligation in rats	Intrathecal injection of 30, 100, 300 μg/kg ketamine	Ketamine reduced mechanical allodynia in a dose-dependent manner
Mei, et al. (2011b)	Spinal nerve ligation in rats	Intrathecal injection of 100, 300 μg/kg/day ketamine for 2 days	Ketamine reduced mechanical allodynia without interfering with motor performance
Mei et al. (2010)	Spinal nerve ligation in rats	Intrathecal injection of 10, 100, 1,000 μg/kg ketamine	Ketamine reduced mechanical allodynia in a dose-dependent manner
Burton et al. (1999)	Spinal nerve ligation in rats	Intrathecal injection of 1 mg/kg ketamine at 15 min before injury	Ketamine reduced mechanical allodynia, cold allodynia, and ongoing pain as revealed by the von frey hair, acetone, and cold plate testing, respectively. This decrease in allodynia was maintained for at least 2 weeks
Cohen et al. (2009)	Spinal nerve ligation in rats	Intrathecal injection of 1 mg/kg ketamine	Ketamine reduced mechanical and cold allodynia for 2 weeks
Wang et al. (2011)	Spared nerve injury in rats	Intraperitoneal injection of 10 mg/kg ketamine	The single injection of ketamine did not alter spared nerve injury-induced hypersensitivity. However, it did reduce spared nerve injury-associated depression-like behaviors
Cohen et al. (2006)	Spared nerve injury in rats	Intraperitoneal injection of 10 mg/kg ketamine	Ketamine did produce significant analgesic effects
Swartjes et al. (2011)	Spared nerve injury in rats	Intravenous infusions of 3 mg/kg/hour ketamine for 3 h for 5 consecutive days (9 mg/kg/day for 5 days)	Ketamine infusion reduced mechanical allodynia with maximum relief occurring at postoperative week 2
Cohen et al. (2004)	Spared nerve injury in rats	Intravenous infusion of 3 mg/kg/h for 3 h daily for five consecutive days (9 mg/kg/day for 5 days)	Ketamine decreased mechanical and cold allodynia for 3–6 weeks after the first injection
Vega-Avelaira et al. (2012)	Spared nerve injury in rats	Subcutaneous injection of 1, 10, or 20 mg/kg ketamine	Ketamine reduced mechanical nociceptive thresholds. At the highest (20 mg/kg) dose the threshold was returned to control levels
ND et al. (2019)	Chronic constriction injury in rats	Intraperitoneal injection of 30, 40, or 50 mg/kg ketamine	Ketamine reduced heat hyperalgesia at 2 and 3 h after injection and reduced mechanical allodynia at 1 h after injection
Vissers et al. (2006)	Chronic constriction injury in rats	Intraperitoneal injection of 40 mg/kg ketamine	Ketamine reduced cold allodynia at 1 h
Christoph et al. (2006)	Chronic constriction injury in rats	Intravenous injection of 4.64 mg/kg ketamine	Ketamine (4.64 mg/kg) reduced cold allodynia for more than 3 h
Pelissier et al. (2003)	Chronic constriction injury in rats	Subcutaneous injection of 12.5, 25, 50, or 100 mg/kg ketamine	The ED25 and the 95% confidence limit (in parentheses) of ketamine were 31.8 (23.9–42.2) mg/kg
Lim et al. (2013)	Chronic constriction injury in mice	Intrathecal injection of 3, 10, 30, or 100 µg ketamine	The 3 and 10 µg dose of ketamine was not effective on neuropathic pain while the 30 and 100 µg showed significant analgesia
Claudino et al. (2018)	Constriction of the infraorbital nerve in rats	Intranasal administration of 0.5 or 1 mg/kg ketamine	Intranasal ketamine at 0.5 mg/kg reduced the heat hyperalgesia 4 days post-injury and at 1 mg/kg attenuated the mechanical hyperalgesia at 14 days post-injury
Staff (2015)	Complete spinal cord transection in rats	Intraperitoneal injection of 50 mg/kg ketamine	Acute treatment with ketamine reduced mechanical allodynia for 1 h
Diabetic neuropathic pain			
Mak et al. (2015)	Streptozotocin-induced diabetic neuropathy in rats	Subcutaneous infusion of 1) ketamine 20 mg/kg/day + morphine 20 mg/kg/day for 5 days, 2) ketamine 20 mg/kg/day for 5 days	Each treatment of 1) and 2) reduced heat hyperalgesia for 12 and 4 weeks, respectively. The ketamine-treated rats also demonstrated opioid-sparing effects
Claudino et al. (2018)	Streptozotocin-induced diabetic neuropathy in rats	Subcutaneous infusion of 20 mg/kg/day ketamine for 5 days	The 5-days ketamine infusion showed anti-hyperalgesia for up to 4 weeks post-treatment
Other neuropathic pain			
Zhou et al. (2018)	Complete Freund’s adjuvant injection in rats	Intraperitoneal injection of 10 mg/kg and then 20 mg/kg for 8 days	Ketamine reduced aversive effects on day 2 after the first ketamine injection
Ward and Standage (2003)	Arthritic pain in rat by CFA	Subcutaneous injection of 2, 10 mg/kg ketamine x20 doses in 4 weeks	Ketamine reduced arthritic pain starting on week 2 for 10 mg/kg dosing and week 3 for 2 mg/kg dosing
Tajerian et al. (2015)	Distal tibia fracture and casting in mouse	Subcutaneous injection of 2 mg/kg/day ketamine for 7 days starting week 7 after fracture	Ketamine reduced allodynia for 4 weeks after termination of ketamine
Liman et al. (2015)	Ischemia and reperfusion in rats	Intraperitoneal injection of 100 mg/kg ketamine	Ketamine reduced mechanical and cold allodynia
Sasaki et al. (2008)	Inoculation of herpes simplex virus type 1	Intraperitoneal injection of 50 mg/kg ketamine	Ketamine reduced allodynia on day 7 after inoculation

### Acute Pain

The use of ketamine have been reported in various acute pain animal models including writhing, hot plate, formalin and tail-flick test in mice and rats. In various models Ketamine was administered by systemic administrations (subcutaneous injection and intraperitoneal injection) or local administrations (intrathecal injection and local injection into paw).

Takahashi et al. reported that subcutaneous injection of ketamine (15, 30 mg/kg) promoted dose-related analgesia in both the acetic acid-induced writhing and hot plate tests in mice. Analgesia was not affected by pretreatment with naloxone (10 mg/kg).

Hillhouse and Negus reported that intraperitoneal injection of ketamine (1.0–10.0 mg/kg) blocked lactic acid-stimulated stretching in rats but failed to block lactic acid-induced depression in rats. In addition, higher doses of ketamine (10 mg/kg) depressed behaviors including lactic acid-stimulated stretching and lactic acid-induced depression.

Finck et al. showed that the subcutaneous injection of ketamine (20, 25, 30 mg/kg) reduced the number of writhes in the morphine pellet-implanted mice using the abdominal constriction test. On the other hand, ketamine was found to be much less effective as an analgesic agent in the morphine tolerant mice. The data indicated a possible cross-tolerance between morphine and ketamine.

Millan and Seguin reported that in the formalin-induced licking behavior by intraplantar injection of formalin in mice and intraperitoneal injection of ketamine blocked both the early and late phases. The doses of ketamine used were 6.0 mg/kg for early phase and 7.7 mg/kg for late phase.

Bulutcu et al. reported that intraperitoneal injection (1, 5 or 10 mg/kg) or intrathecal injection (10, 30 or 60 mcg/mouse) of ketamine produced dose-dependent antinociceptive effects in the acetic acid-induced writhing and formalin tests but not in the tail-flick nor in hot-plate tests in mice.

Lastly, Sawynok and Reid reported that local pretreatment with 10–1,000 nmol ketamine via subcutaneous injection into the dorsal surface of the hind paw had no effect on phase 2 flinching behaviors and phase 2 biting/licking behaviors produced by both 1.5% and 5% formalin. In addition, ketamine produced no effect on phase 1 behaviors. In addition, systemic intraperitoneal injection of ketamine (10, 30, 60 mg/kg) generally had no effect on phase 1 and 2 flinching behaviors.

## Clinical Evidence of Ketamine Efficacy

For much of the drug’s lifespan ketamine has been used primarily as a dissociative anesthetic and a pediatric analgesic for acute pain in the emergency department. However, more recently there has been renewed interest in the drug for potential treatment of refractory pain, substance abuse, and treatment resistant depression. Indeed, both intranasal and oral ketamine have recently garnered FDA approval for the treatment of depression (Gautam et al., 2020).

While the latest Cochrane Review from 2017 found insufficient evidence to recommend Ketamine as an adjunctive therapy in cancer pain, significant clinical evidence is building that demonstrates ketamine as a potent analgesic with a limited adverse event profile (Bell et al., 2017).

### Intravenous Infusions for Chronic Pain

One of the first applications of ketamine for analgesia was via intravenous infusion due to the ability to avoid first-pass metabolism and the well-controlled nature of administration. Additionally, these infusions typically occurred in inpatient settings allowing the healthcare team to monitor for adverse conditions and track treatment efficacy.

A meta-analysis including a total of 211 patients in seven different studies revealed a significant analgesic effect for intravenous ketamine infusions in both neuropathic and non-neuropathic pain conditions when compared to placebo. The average infusion duration for these patients was 5 h reaching a median ketamine dose of 0.35 mg/kg. In all seven studies, maximum analgesic effect was observed between 48 h and 2 weeks post-infusion. Magnitude of pain reduction remained significant until 4 weeks post-infusion with some effect seen as far as 8 weeks out. Efficacy of ketamine was not significantly different between pain conditions or classifications. The studies also reported no efficacy difference between ketamine as a standalone or adjuvant therapy. In all seven studies, maximum analgesic effect was observed between 48 h and 2 weeks (Orhurhu et al., 2019).

Taking the above meta-analysis into consideration, ketamine shows significant promise for the treatment of a wide variety of chronic pain conditions, including neuropathic and non-neuropathic. Furthermore, due to the long-acting nature of ketamine’s analgesia this treatment can be offered outpatient with visits to pain management only required as frequently as infusions are needed.

Case studies also support the use of ketamine as a third-line agent in intractable cancer pain. In 12 cancer patients with intractable pain, ketamine infusions at a rate of 1.5 mg/kg/day reduced total daily morphine use by 50% after patients were sent home with ketamine/morphine pain pumps. Side effects were minimal and only occurred with the initial testing dose (Lossignol et al., 2005).

### Oral and Nasal Ketamine for Chronic Pain

In contrast to infusions of ketamine, oral and nasal formulations are commonly administered without direct physician supervision and are therefore more desirable for management of long-term pain conditions. While they require significantly higher doses due to extensive metabolism, oral administrations have also proven effective in providing analgesia.

Two studies investigating oral ketamine analgesia both showed at least mild pain reduction. One study with a daily dose of 2 mg/kg ketamine reduced or abolished pain in two-thirds of patients while one-half of patients reported some adverse event. Only half of these patients decided to stop the ketamine therapy. Interestingly, the incidence of adverse events was lower in patients concomitantly taking opioids (Marchetti et al., 2014). The second study also demonstrated significant pain score reduction with the most frequent adverse event reported being increased drowsiness (Kannan et al., 2002).

In addition to reduction of pain, oral ketamine has also shown promise in countering Opioid-induced Hyperalgesia (OIH). Patients administered oral ketamine (0.5 mg/kg every 12 h) had significantly lower oral morphine consumption than those in a control group. Indicating that ketamine has some analgesic and opioid tolerance sparing ability. Additionally, the ketamine patients reported decreased somnolence perhaps due to the decreased opioid dosing (Lauretti et al., 1999).

Intranasal administration of ketamine, although now more associated with treatment of depression, has also been tried for treatment of cancer pain. After treatment with intranasal ketamine using a nasal spray pump, 65% of breakthrough cancer pain patients achieved a Numerical Pain Intensity Scale (NPIS) score that was at least 40% lower than pre-treatment levels compared to 20% of placebo patients achieving the same metric (*p* = 0.007) (Carr et al., 2004). Other studies of intranasal ketamine are scarce, however with the emergence of FDA approved intra-nasal ketamine solutions for other conditions it is possible that more studies will be undertaken in this area.

### Ketamine as a Topical Agent

For patients in whom systemic ketamine administration via oral or IV routes is not desirable administration of the drug as a topical agent may be possible. As with many drugs, this route provides the benefit of keeping plasma concentrations and therefore potential side effects at a minimum.

It is important to note that In addition to the central nervous system, NMDA receptors are widely present in the axons of the peripheral nervous system. In neuropathic and inflammatory pain conditions, topical ketamine alleviates pain by down regulating the upregulated NMDA, AMPA and kinate receptors (Coggeshall and Carlton 1998; Carlton, 2009).

One of the most frequent disorders that is treated with a ketamine topical is Chronic Regional Pain Syndrome (CRPS), a type of chronic neuropathic pain. Ketamine creams, both a 10% Ketamine only and a combination of ketamine and several other analgesics have been shown effective in reducing pain measures, tactile allodynia and Visual Analog Scale (VAS) pain score, in these patients in as little as 30 min and in a fraction of patients similar to systemic treatment (Heir et al., 2008; Finch et al., 2009). These studies also demonstrated that systemic levels of ketamine remain undetectable (Finch et al., 2009).

Others have also demonstrated that topical ketamine and amitriptyline cream is effective in other pain conditions such as postherpetic neuralgia (Lynch et al., 2005; Everton et al., 2007).

While these studies are promising, there are also several studies that seem to contradict their results. Many of the same topical applications mentioned above have been tested and shown no effect in other studies (de Barros et al., 2012; Gewandter et al., 2014; Lynch et al., 2003, Lynch et al., 2005). However, these studies did confirm that the adverse event profile of topical ketamine is minimal. Therefore, while the evidence for topical ketamine is not as strong as other routes of administration it may still be worth trying for some patients due to low risk. A tabulated form of the clinical evidence discussed throughout the preceding sections can be found in Table 2.

**TABLE 2:** Compiled clinical evidence supporting ketamine’s analgesic effects.

Authors	Pain condition	Study type	Ketamine treatment	Sample size	Results	Comments
Intravenous ketamine infusion						
Schwartzman et al. (2009)	Chronic regional pain syndrome (CRPS)	Double-blind placebo controlled	Maximum of 0.35 mg/kg/h × 4 hr × 10 days	19	Intravenously ketamine administered via outpatient setting significantly reduced several pain parameters	Avg. Pain decrease 27% vs 2% placebo. Co-administered w/clonidine and midazolam
Orhurhu et al. (2019)	Mixed neuropathic and non-neuropathic	Meta-analysis	Varies, median 0.35 mg/kg/h for 5 h	211	Ketamine has significant analgesic benefit up to 2 weeks after cessation of administration. High-dose ketamine significantly more effective than low-dose	No significant difference in ketamine efficacy based on pain condition
Lossignol et al. (2005)	Intractable cancer pain	Case series	1.5 mg/kg/day ketamine	12	Ketamine infusion improved pain controlled and reduced total daily morphine use by an average of 50%	Infused concomitantly w/morphine. Only moderate side effects (dizziness)
Oral ketamine						
Rigo et al. (2017)	Refractory neuropathic pain	Double-blind active controlled	3 mg methadone OR 30 mg ketamine OR 3 mg methadone +30 mg ketamine (3× daily)	14	Ketamine is superior to methadone and methadone/ketamine combination in treating neuropathic allodynia	All treatments reduced VAS score by at least 40%. No differences in burning/shooting pain reduction
Lauretti et al. (1999)	Cancer pain	Multi-arm treatment	0.5 mg/kg 2× daily	15	Low-dose ketamine significantly reduced daily morphine consumption and reduced somnolence in patients suffering from cancer pain	No somnolence reported with oral ketamine. Lower rates of adverse effects compared to morphine, nitroglycerin, and dipyrone groups
Kannan et al. (2002)	Neuropathic cancer pain	Case series	0.5 mg/kg 3× daily	9	Seven of nine patients report decrease in pain score ≥3 compared to when solely taking maximally tolerated opioid dose	Adverse effects: Nausea (×4), loss of appetite (×2), sleepiness (×8, improved to ×3). Three pts withdrew due to effects
Marchetti et al. (2014)	Intractable cancer pain	Case series	1.5–3 mg/kg daily (varied schedules)	51	Pain reduced or abolished in 2/3 of patients with ketamine treatment	Patients on opioid therapy had lower failure rate of ketamine treatment than non-opioid group
Intranasal ketamine						
(Carr et al. (2004)	Breakthrough cancer pain	Double-blind crossover	10–50 mg (1–5 sprays)	10	After treatment with intranasal ketamine, 65% of breakthrough cancer pain patients achieved an NPIS score that was at least 40% lower than pre-treatment levels compared to 20% of placebo patients	No reports of auditory or visual hallucinations. Adverse effects: Change of taste (×4), rhinorrhea (×1), BP elevation (×2)
Topical ketamine						
Finch et al. (2009)	Chronic regional pain syndrome	Double-blind placebo controlled	10% ketamine cream	20	KET cream reduced CRPS associated allodynia and hyperalgesia within 30 min of administration	Systemic ketamine concentrations below detectable limit
Gammaitoni et al. (2000)	CRPS, lumbar radiculopathy, post-herpetic neuralgia	Case series	1% ketamine cream	5	Reduction in pain scale ranging from 53–100% 15 min after application of cream	No side effects reported
Heir et al. (2008)	Orofacial neuropathic pain	Case-control series	4% KET, 4% carbamazepine, 1% ketoprofen, 4% gabapentin	39	41% of patients had a decrease in pain level of at least 30%. This proportion is similar to systemic and systemic + topical treatment modalities	Pain relief lower in magnitude than systemic treatments, but shorter time to onset.
Everton et al. (2007)	Post-herpetic neuralgia	Double-blind placebo controlled	2% ketamine +4% amitriptyline	118	46% of AmiKet patients reported a decrease in daily pain score of at least 30% compared to 19% in placebo group	Only 5% of patients had detectable systemic ketamine or amitriptyline levels

### Ketamine in the Pediatric Population

Documented use of ketamine for analgesia in the pediatric population is scarcer, the instances which we do have are promising. Ketamine therapy has been shown effective in a variety of pain conditions in the pediatric population and with a minimal adverse event profile similar to its general population use. Initial animal studies raised concern regarding the toxicity of ketamine to developing neuronal cells. However, these worries have been lessened as more recent studies have demonstrated that the developmental stage at which these animal studies took place correlates to a pre-birth period in humans.

A case study of 12 children treated 3x daily with oral ketamine demonstrated results similar to those in adults. Five of the 12 patients experienced significant improvement in pain scores that lasted up to four weeks after the ketamine treatment. Two patients eventually treated at the highest dose of 1.5 mg/kg/dose experienced dose-limiting toxicities and ceased treatment (Bredlau et al., 2013).

In another case study of 11 children ranging in age from 3–17 years old with inadequate pain control despite opioid escalation 73% of patients reported subjectively improved pain scores and total opioid consumption was decreased by 28%–100%. These patients were receiving lorazepam concurrently to prevent psychiatric side effects (Finkel et al., 2007).

Likewise, a 13 year-old patient with refractory pain following pancreatitis was treated with hydromorphone until reaching a total daily morphine equivalent of 1,375 mg and being admitted to the ICU. Following a sub-anesthetic administration of IV ketamine average 5 days pain score was rapidly reduced from 8.3 to 4.4 while OME requirements were reduced to 375 mg. The patient experienced no adverse effects from the drug (Mulder et al., 2018).

Finally, a single case report detailing a 14 year old patient with pain refractory to other treatments was admitted to the PICU and administered an infusion of ketamine at 7 μg/kg/minute for 24 h a day. Not only did this patient report a 70% decrease in total pain score, but also a decrease in depressive symptoms. Due to the success of this treatment, the patient was retreated in the same manner when presenting again 5-months later (Weber et al., 2018). The mitigation of depressive symptoms in pain conditions could potential serve as a niche application for ketamine in the future. Clinical evidence supporting the use of ketamine as an analgesic specifically relating to the pediatric population can be found in Table 3.

**TABLE 3:** Compiled evidence of ketamine’s analgesic effect in the pediatric population.

References	Pain condition	Study type	Ketamine treatment	Sample size	Results	Comments
Bredlau et al. (2013)	Chronic pain	Prospective case series	Oral (3x daily). 25–1.5 mg/kg/dose	12	Five of twelve children reported experienced marked improvement in pain scores lasting >4 weeks off ketamine treatment	Two children forced to withdraw treatment due to dose-limiting toxicities
Finkel et al. (2007)	Cancer pain	Retrospective case series	Infusion 0.1–1 mg/kg/h	11	Sub-anesthetic dosages of ketamine infusion resulted in subjectively improved pain control and a reduction in required opioid dosage ranging from 28 to 100%	Administered with lorazepam, no psychotropic effects reported. Patients more awake and alert than on opioids alone
Mulder et al. (2018)	Acute pain (pancreatitis)	Case report	Infusion 2 μg/kg/min	1	Rapid reduction in pain score from 8.3 to 4.4. Total daily oral morphine equivalent reduced from 1,375 to 375 mg/day	N/A
Weber et al. (2018)	Neuropathic pain	Case report	Infusion 7 μg/kg/min	1	Self-reported 70% reduction in pain. Pain relief lasted five months after initial ketamine infusion	Suicidal ideation also resolved. Patient retreated at 5-months mark with same results

### Contraindications

While ketamine is regarded as safe for the general population, the drug may be contraindicated or at least require special precautions for some sub-populations. The first of these is in patients who have a history of schizophrenia, psychotic symptoms, or Post-Traumatic Stress Disorder (PTSD). Ketamine has been shown to exacerbate and/or elicit psychotic symptoms in these patients and its use is therefore non-desirable (Lahti et al., 2001; Xu et al., 2015). When used in this population, strong consideration should be given to the use of benzodiazepines to help prevent development of psychotic symptoms (Krystal et al., 1999; Sinner and Graf, 2008).

Another population that may require special caution is patients with cardiovascular or hepatic compromise. Ketamine has been shown to produce increased cardiac stimulation which may be harmful to patients with previous cardiovascular disease (Dahan et al., 2009). Likewise, ketamine is known to be hepatotoxic, especially with chronic use, and exhibits a rise in liver function tests (LFTs) in up to 80% of patients (Kiefer et al., 2008).

Finally, ketamine is widely considered as contraindicated in patients less than 3 months of age due to complications in maintaining a patent airway (Dolansky et al., 2008). While some may consider this contraindication to extend out to 12 months of age, however there is evidence of use in this population with no adverse events (Green and Krauss, 2004).

### Summary of Clinical Evidence

The above studies provide significant evidence that ketamine can be safely utilized for its analgesic properties in sub-anesthetic doses. Intravenous and oral/intranasal ketamine proved effective in a wide range of pain conditions including neuropathic pain, CRPS, and acute pain exacerbations. While hypothesized that ketamine may provide special benefits in neuropathic pain, the studies which explicitly examined this found no difference in efficacy based on pain condition. The dosages which provided effective analgesia were also consistent across pain conditions with most studies finding an i. v. infusion of 0.35 mg/kg ketamine effective for analgesia for up to two weeks. For oral ketamine, a daily dose around 1–1.5/kg mg seemed to be the standard and produced effects in >50% of patients.

Topical ketamine has much less supporting evidence for its use but several reports detail efficacy in CRPS with minimal systemic absorption and adverse events.

The average dosage required for anesthesia in patients is 2 mg/kg as a single bolus over 1 min with anesthesia only lasting 5–10 min (White and Kershaw, 2015). Of the i. v. studies referenced the dose nearest to this is a 0.35 mg/kg infusion over 1 h. If normalized to a 1-min bolus, this would equate to less than 0.006 mg/kg/min infusion rate which is clearly well below that required for anesthesia. The difference in the dosages providing analgesia and anesthesia provide a rather large therapeutic window, especially if the analgesic dose can be spread over time.

This therapeutic window is also confirmed for oral ketamine by plasma studies which demonstrate that analgesia is obtained at ketamine concentrations of 0.04 μg/ml when taken orally while anesthetic effects are not observed till a range of 0.64–1.12 μg/ml (White and Kershaw, 2015).

## Adverse Effects and Addiction

Sub-anesthetic ketamine infusions were associated with adverse drug events (hallucination, dysphoria, dizziness, visual disturbance, or sedation) in 29.5% of patients. This occurrence rate was significantly lower (10.3% vs. 37.3%) in patients diagnosed with depression. The researcher’s hypothesis is that increased NMDA-receptor activity seen in depression is protective against psychotic effects of ketamine (Stoker et al., 2019).

### Ketamine Cognitive Effects

Ketamine has long been known to have cognitive side effects that frequently limit its use. In fact, ketamine was known to produce dissociative and psychomimetic effects within years of its discovery (Domino et al., 1965). These cognitive effects are also responsible for the continued abuse of ketamine as a street drug.

One of the most prominent cognitive side effects is the sense of a general “euphoria” and disconnection from their surroundings. In a study of healthy volunteers, ketamine administration produced a discernible change in subjective experience in patients including prominence of distant sounds, feelings of dreaminess, loss of attentiveness, and illusory perception of movements. With an oral dose of 25 mg of ketamine, all patients were able to distinguish a feeling of dreaminess/less attentiveness compared to a when receiving a placebo (Harborne et al., 1996).

In addition to these euphoric feelings, ketamine has also been shown to produce a formal thought disorder and impairments in both working and semantic memory function in healthy volunteers (Adler et al., 1998). In chronic ketamine abusers, these memory impairments have been shown to persist even after abstinence from ketamine suggesting permanent damage to the brain (Niesters et al., 2014).

While the exact mechanism of these cognitive effects is unknown, ketamine has been shown to produce both structural and physiological changes in the brain. One study shows that ketamine produced a focal increase in metabolic activity in the pre-frontal cortex that was associated with development of an acute psychotic state (Breier et al., 1997). Meanwhile in a series of two studies *Lia et al.* showed decreased gray and white matter volumes in the pre-frontal cortex and white matter degeneration in the left temporoparietal lobe (Liao et al., 2010; Liao et al., 2011).

Regardless of mechanism, the occurrence of these well-documented cognitive events has generated research into what psychiatric drugs may be able to prevent them and therefore allow wider use of ketamine. Several of these studies have demonstrated that benzodiazepines, specifically midazolam and haloperidol, reduced undesired psychotic side-effects and nausea associated with ketamine administration (Mathisen et al., 1995; Mercadante et al., 1995).

### Ketamine Cystitis

Due to a high occurrence in chronic ketamine abusers, ketamine cystitis is also a concern when discussing long-term use of ketamine as analgesia. Urinary tract symptoms indicative of cystitis are reported in over 25% of long-term ketamine abusers with occurrence related in a dose and frequency dependent manner. Also of importance is that only 51% of respondents reported improvement in symptoms following cessation of ketamine (Winstock et al., 2012).

The chronic inflammation of the bladder and urinary tract in ketamine cystitis may be due to abnormal increases in neurotrophin seen in bladder tissue exposed to ketamine. Rats taking ketamine have also been observed to have increased expression of iNOS and COX enzymes within bladder tissue. The only conclusive treatment for ketamine cystitis is cessation of the drug although, as mentioned above, some patients with irreversible damage such as thickened bladder walls and hydronephrosis may have symptoms persist and require surgical intervention (J.-F. Jhang et al., 2015).

One unique aspect of ketamine-related cystitis is a significant elevation in serum IgE levels when compared to control patients and those with an acute bacterial cystitis. This elevated IgE was also significantly correlated with pain severity and decreased total bladder capacity (J. F. Jhang et al., 2014). Perhaps this IgE elevation could be a potential therapeutic target for the prevention of ketamine cystitis or even a biomarker for its potential onset.

### Ketamine Hepatotoxicity

Patients administered ketamine infusions for treatment of CRPS showed signs and symptoms of drug-induced liver injury as evidenced by elevated serum AST, ALT, and gamma-glutamyl transferase levels. Frequency of this drug-induced hepatotoxicity caused the authors to end their proposed study earlier than anticipated (Noppers et al., 2011).

Sixteen of twenty (80%) of CRPS patients treated with ketamine infusions for refractory pain experienced transient elevations in lever enzyme (AST, ALT, GGT) levels that decreased back to baseline within 10–14 days following treatment (Kiefer et al., 2008).

### Ketamine Cardiovascular Effects

Isolated human-heart studies have indicated that ketamine acts directly on cardiac myocytes as a concentration-dependent negative inotropic agent. However, due to its central inhibition of norepinephrine reuptake in adrenergic nerves it generally produces an increase in cardiac output via elevations in heart rate, systolic blood pressure, and diastolic blood pressure (Suleiman et al., 2012)**.** In this way, ketamine acts like a sympathomimetic on the cardiovascular system although exceptions are seen with some critically-ill patients.

As evidence of the potential magnitude of this effect, a study of 20 healthy volunteers documented a 40–50% increase in total cardiac output when given intravenous infusion of ketamine at analgesic concentrations (Dahan et al., 2009).

While most examples of ketamine acting as a sympathomimetic involve intravenous infusions (largely due to the drugs use in inducing anasthesia), it is also important for analgesia purposes that the oral rout of administration does not negate this sympathomimetic effect.

A 67-year-old woman introduced to oral ketamine in the hospital for refractory chronic pain developed uncontrolled hypertension (222/124 mmHg). After release, the patient was not immediately followed up with but the hypertension was found to be persistent by her GP until cessation of the oral ketamine (Van Hecke and Guthrie, 2014). These facts may make monitoring of blood pressure and other cardiovascular parameters more important if using ketamine as a long term analgesic, especially in patients who may suffer from cardiovascular disease.

### Ketamine Neurotoxicity

Concern of ketamine’s potential neurotoxicity arise from animal studies demonstrating that ketamine administration in rats results in dose-related and time-related increases in neuronal apoptotic cell death in the frontal cortex and other areas of the brain during development (Zou et al., 2009).

However, the validity of these studies to humans is frequently challenged. Criticism of the above (and many other ketamine neurotoxicity studies) argues the ketamine neurotoxicity observed in rat studies is not clinically relevant as the stage at which the rat studies take place is during the CNS growth spurt corresponding to 22–26 weeks gestation in humans, therefore humans would never be exposed to ketamine as ketamine is widely regarded as contraindicated <3 years of age (Green and Coté, 2009).

Even so, long-term treatment with ketamine does come with some documented potential for neurotoxicity. Specifically, it has been found that ketamine treated neurons are more susceptible to damage as a result endogenous glutamate release. This increased susceptibility is due to recruitment of more NMDA receptors following prolonged blockade. When activated this increased number of receptors lead to calcium overload by exceeding the buffer capacity of mitochondria and interfering with the electron transport chain resulting in production of ROS (C. Wang et al., 2017).

### Ketamine Addiction

Ketamine and other members of the PCP-derivative family have a long history of drug abuse, especially by street drug users. Ketamine has even earned the street name of “Special K” due to its widespread use. Ketamine abusers are ultimately targeting the dissociative and hallucinogenic effects of the drug which are described as euphoric even in ketamine naïve subjects (Morgan et al., 2004; Domino, 2010).

The mechanism behind this addiction potential is believed to be a hijacking of the brains dopamine-based reward system. Subanesthetic doses of ketamine acutely increased extracellular concentration of dopamine in rat striatum and prefrontal cortex. Some human PET imaging also suggests that this occurs in humans. This mechanism that mimics the brain’s reward system associated with addiction in other pharmaceuticals (Moghaddam et al., 1997; Smith et al., 1998).

While therapeutic doses are generally less than street-use doses, several studies detailed below detail that even when used medically ketamine maintains addictive potential. When healthy ketamine-naïve subjects were infused with subanesthetic doses of ketamine they reported “liking” the feeling induced by ketamine and wanting more doses. In a group receiving higher doses of ketamine these cravings were relatively reduced while those on the lower dose infusions reported increasing desire for more of the drug (Morgan et al., 2004).

Another example of ketamine’s addictive potential comes from a 50-year-old anesthesia nurse who was suffering from Major Depressive Disorder (MDD). The nurse initially began self-medicating with weekly IM injections of ketamine. After weeks of self-medication, the patient began to develop significant tolerance to ketamine and had to stepwise increase both dose and frequency of the ketamine. Along with tolerance, the patient also developed significant ketamine addiction. When ketamine was stopped, patient began suffering intense craving and depressive episodes (Bonnet, 2015).

While the research on ketamine’s addictive potential when used medically is somewhat limited, it should not be overlooked and both physicians and patients should be aware of and discuss this potential before adoption of ketamine-based therapies.

## Limitations of Studies

While ketamine has been available for half a century, it has not been widely used in the medical community for decades. This has led to limited recent literature on its use, especially as an analgesic. Much of the information available on the use of ketamine for chronic pain relief is within case reports/series with relatively small sample sizes.

Unfortunately, these limited numbers provide us with limited power to determine the true efficacy of ketamine in chronic pain conditions. This may be further exacerbated by publication bias in favor of publishing reports in which ketamine is effective. Reliance on case reports also leads to the possibility of treatment effect as almost none of these reports make use of placebo or non-active controls. These shortfalls make it evident that more large-scale placebo controlled clinical trials are necessary before being able to make a statistically strong conclusion on ketamine as a long-term analgesic.

Another concern in some of the referenced studies is the possibility of confounding variables due to the large number of medications/treatments patients are often placed on prior to initiating ketamine therapy. Ketamine is currently used primarily as third-line analgesia and therefore patients are often on high-dose NSAIDs and opiates prior to trial of ketamine. This makes it hard to distinguish the singular effect of ketamine on pain relief, especially in the setting of opioid induced hyperalgesia.

Research into the adverse event profile of ketamine has also been hampered by the infrequent use of the drug in monitored clinical settings. While the papers referenced in this article provide information on the short-term development of side effects, less is known about the long-term effect of ketamine treatment. Most of what we do know about the long-term development of problems such as ketamine cystitis and hepatotoxicity has been on subjects who used ketamine as a recreational street drug and at much higher doses than used for analgesia. This raises questions as to if patients treated medically with ketamine would experience the same appearance of these symptoms.

## Discussion

In this article, we present ketamine as a therapy for cancer and chronic pain that is intractable to well-established therapeutics such as opioids. While ketamine has long been known as a dissociative anesthetic, its use as an analgesic agent in non-anesthetic doses is much more recent.

Ketamine’s analgesic effects are believed to be an effect of NMDA-receptor blockade which decreases central neuronal excitability and therefore conduction of pain impulses. However, ketamine may also have other beneficial mechanisms of action when used to treat neuropathic pain such as the inhibition of microglial activation and neuronal inflammation as discussed above and demonstrated in several animal studies.

Ketamine has repetitively been shown to reduce pain scores and subjective measures of pain. While the use of topical ketamine alone does not have strong supporting evidence, its use in multi-agent creams has been effective in some pain conditions.

Use of ketamine has classically been associated with dissociative effects, however several recent studies have demonstrated that at sub-anesthetic dosages the occurrence of psychomimetic effects in the general population is minimal. Furthermore, the risk of these adverse events can be further reduced with pre-medication of benzodiazepines. A unique aspect of ketamine treatment in chronic pain patients is that it seems to counteract opioid-induce hyperalgesia and not therefore not only improves pain management but does so while simultaneously reducing a patient’s required total daily morphine equivalent. In some instances, this has been reported to improve quality of life while in others it serves to improve respiratory and hemodynamic stability.
